# Automated White Matter Hyperintensity Detection in Multiple Sclerosis Using 3D T2 FLAIR

**DOI:** 10.1155/2014/239123

**Published:** 2014-07-22

**Authors:** Yi Zhong, David Utriainen, Ying Wang, Yan Kang, E. Mark Haacke

**Affiliations:** ^1^School of Sino-Dutch Biomedical and Information Engineering, Northeastern University, Shenyang, Liaoning 110004, China; ^2^Magnetic Resonance Innovations Inc., 440 E. Ferry Street, Detroit, MI 48202, USA; ^3^Department of Biomedical Engineering, Wayne State University, Detroit, MI 48201, USA; ^4^Magnetic Resonance Imaging Institute for Biomedical Research, 440 E. Ferry Street, Detroit, MI 48202, USA

## Abstract

White matter hyperintensities (WMH) seen on T2WI are a hallmark of multiple sclerosis (MS) as it indicates inflammation associated with the disease. Automatic detection of the WMH can be valuable in diagnosing and monitoring of treatment effectiveness. T2 fluid attenuated inversion recovery (FLAIR) MR images provided good contrast between the lesions and other tissue; however the signal intensity of gray matter tissue was close to the lesions in FLAIR images that may cause more false positives in the segment result. We developed and evaluated a tool for automated WMH detection only using high resolution 3D T2 fluid attenuated inversion recovery (FLAIR) MR images. We use a high spatial frequency suppression method to reduce the gray matter area signal intensity. We evaluate our method in 26 MS patients and 26 age matched health controls. The data from the automated algorithm showed good agreement with that from the manual segmentation. The linear correlation between these two approaches in comparing WMH volumes was found to be *Y* = 1.04*X* + 1.74  (*R* ^2^ = 0.96). The automated algorithm estimates the number, volume, and category of WMH.

## 1. Introduction

Multiple sclerosis (MS) is considered an autoimmune inflammatory demyelinating disease affecting the central nervous system. It manifests as white matter hyperintensities (WMH) as seen on T2-weighted imaging (WI) using magnetic resonance imaging (MRI). The high signal intensity lesions are a hallmark of MS and are believed to represent inflammation associated with the disease. It has been reported that WMH lesion volume and brain atrophy are independent risk factors for conversion to MS [1, 2]. After diagnosis, MS patients are followed longitudinally and often receive MR imaging multiple times to monitor lesion development. This necessitates the need for a tool which radiologists, neurologists, and MS researchers can use to quantify parenchymal WMH accurately and efficiently.

Although T2-weighted imaging remains important in imaging MS patients, its ability to delineate WMH is usually hampered by the fact that WMH and cerebral spinal fluid (CBF) are both bright. This drawback is overcome by using T2-weighted fluid attenuated inversion recovery (FLAIR) which suppresses the CSF signal and yet maintains good contrast between the lesions and the white matter (WM). Despite this advantage, several challenges remain for quantifying WMH with FLAIR images, including (1) decreased contrast between gray matter (GM) and WM especially in elderly patients; (2) the major spatial variations in the MR images caused by variable radiofrequency response across the brain (this is referred to as the bias field); and (3) background noise which makes it difficult to separate lesions from white matter. Our goal in this paper is to create an automated white matter lesion detection algorithm which is capable of estimating the volume of WMH accurately, allowing for future ease of use in a clinical setting.

Many automatic WMH quantification techniques for MS lesion detection are reported in the literature [3–7]. Most involve the use of various MRI techniques including T1WI, T2WI, spin density WI, and 2D FLAIR. Although using multiple data sets provides more objective information to identify the WMH, it requires more time and co-registration processes are needed. Comparing 2D FLAIR image and 3D FLAIR images, the latter provide the best resolution, signal-to-noise, and contrast-to-noise and minimize partial volume effects for detecting WMH [8, 9]. Our goal is to use only 3D T2 FLAIR images to automate the extraction of brain volume, CSF volume, ventricle volume, WMH count, and WMH volume and to automate the categorization of the WMH based on their locations into either deep WM hyperintensities (DWMH) or periventricular hyperintensities (PVH).

## 2. Materials and Methods

### 2.1. Data Acquisition

Twenty-six (26) MS patients (age range: 27–51 years, obtained from Synergy Health Concepts, CA) with a diagnosis of relapsing-remitting multiple sclerosis (RRMS) and twenty-six (26) age matched healthy controls (obtained from Wayne State University, MI) were imaged using 3T Siemens scanners (Siemens, Erlangen, Germany). A TRIO scanner was used at Synergy Health Concepts while a VERIO scanner was used at Wayne State University. Institutional review board approval was obtained for this study from both locations and all subjects signed informed consent. MS patient and volunteer data were collected using a 12-channel head and neck coil arrangement at both sites. Both scanners used the same 3D T2 FLAIR sequence parameters. The imaging parameters for the 3D T2 FLAIR sequence were repetition time (TR) = 6000 ms, echo time (TE) = 396 ms, inversion time (TI) = 2200 ms, flip angle (FA) = 120°, *Nx* × *Ny* = 512 × 512, field-of-view (FOV) = 256 mm × 256 mm, in-plane resolution = 0.50 mm × 0.50 mm, and slice thickness = 1.0 mm.

### 2.2. Data Processing

**Figure 1:**
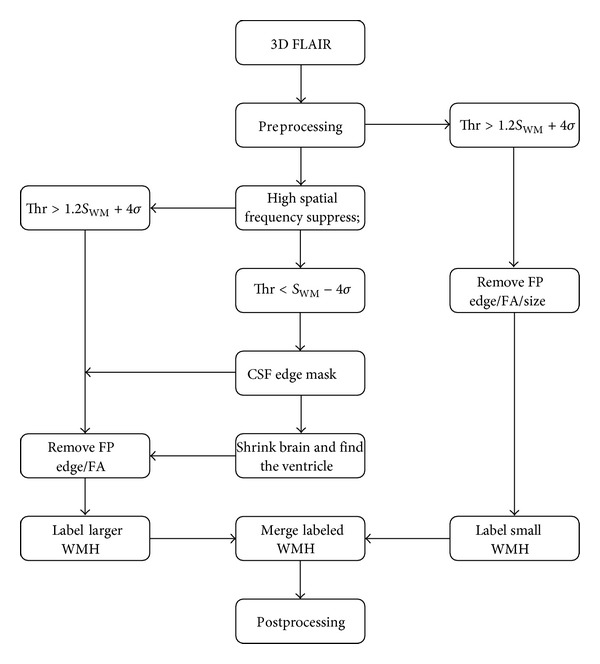
Overall work flow of the automatic quantification of WMH using 3D FLAIR images.

Two novel concepts are introduced to separate WM from the surrounding tissue. One is a high spatial frequency suppression method to remove false GM boundary contributions and the other is a shape-dependent approach to segment WMH from other confounding contrast changes, since conventional threshold segmentation methods contain numerous false positives. The overall workflow, as illustration of Figure 1, our proposed method consisted of seven steps: (1) skull stripping for the removal of the nonbrain tissue; (2) bias field correction for RF field inhomogeneity; (3) CSF sulcus and ventricle segmentation; (4) removal of high spatial frequency GM areas (with the receiver operating characteristic [ROC] curve being used to determine the Hanning high pass window size and other relevant parameters) for WMH segmentation; (5) small lesion detection, for lesions less than 0.025 mL; (6) estimating the lesions volume; and (7) automatic labeling of the lesions as DWMH or PVL. Those lesions with a distance less than 3.0 mm to the edge of the ventricle were considered PVH, while all others were considered DWMH. We describe below the detailed implementation of each step.

#### 2.2.1. Preprocessing: Skull Stripping

In order to apply skull stripping to the 3D T2 FLAIR images, we developed a moment of inertia structure tensor method, which is used for brain surface detection, combined with local morphology to remove nonbrain tissue (previous work in [10]).

#### 2.2.2. Preprocessing: Bias Field Correction

For the bias field correction, a modified homodyne high pass filter approach similar to that used in phase processing for susceptibility weighted imaging [11, 12] and later proposed by Axel et al. for magnitude imaging [13] was applied via

(1) $$\begin{array}{c} u\left(x\right)=\frac{v\left(x\right)}{b\left(x\right)}=v\left(x\right)\cdot \frac{\kappa }{\text{LPF}\left(v^{\prime }\left(x\right)\right)}, \end{array}$$

where *u*(*x*) is the inhomogeneity free image, *v*(*x*) is the original image, *b*(*x*) is the bias field estimate image, and *v*′(*x*) is a filtered, dilated version of *v*(*x*) as described next. LPF stands for low pass filter, while *κ* is a constant based on the most populated signal (the mode) in the brain. The idea is to use the LPF version of *v*(*x*) to estimate low spatial frequency bias field effects. However, this approach itself produces edge effects wherever there are high contrast regions in the brain or at the boundary of the brain. To alleviate this problem, we modified the original image *v*(*x*) to smooth the image. First, we used dilation to extrapolate the brain *v*(*x*) in order to reduce high spatial frequency edge artifacts. In order to get smooth edge expansion, we used a 3 × 3 template to do grayscale dilation over 20 iteration for the brain edge expansion. This new image is then referred to as *v*′(*x*). Finally, the bias field image was estimated by low pass filtering of *v*′(*x*). The original image *v*(*x*) is then normalized to the LPF *v*′(*x*), which is then multiplied by *κ* as shown above.

#### 2.2.3. CSF and Ventricle Extraction

Once the brain has been isolated, the predominant three tissues remaining (in the order of decreasing signal intensities) are GM, WM, and CSF. If there is sufficient contrast between WM and CSF, then we can use the Otsu [14] algorithm to determine statistically the best threshold to remove the pixels with low CSF signal. A binary mask is generated for any value lower than the threshold and the resulting image is then considered as the CSF mask image. Because of the fact that CSF occupies both the subarachnoid space and the ventricle, based on the anatomic connectivity of the subarachnoid and ventricular systems, we applied a large scale 3D erosion of the brain image and multiplied it by the CSF mask image; for the 3D erosion template size we use a ball radius of 50 pixels about 25 mm to make sure we remove most CSF surrounding the brain. The largest remaining connected volume was labeled as the ventricular CSF.

#### 2.2.4. WMH Segmentation with High Spatial Frequency Suppression

White matter lesions have higher intensity and generally clear boundaries relative to the surrounding WM. Unfortunately, in the 3D T2 FLAIR images, the GM can also present with high signal intensity, especially in elderly people. This leads to numerous false positives when attempting to isolate WMH. However, GM structures tend to be narrow; thus they have predominantly high spatial frequency components. If we removed these components, which often tend to mimic lesions in their signal intensities, it may be possible to eliminate many of these false positives. To accomplish this, we created a new low pass filtered image defined via

(2) $$\begin{array}{c} u^{\prime }\left(x\right)=u\left(x\right)-\lambda \cdot \text{HP}\left(u\left(x\right)\right), \end{array}$$

where *u*(*x*) is the original image, HP(*u*(*x*)) is the high pass filtered version of *u*(*x*), and *λ* is a constant to be determined. There are three parameters that affect the lesion segmentation result: the window size of the high pass filter, the constant *λ*, and a WMH threshold value. This threshold is chosen to be 1.2 · S_WM_ + 4 · *σ*_WM_, where S_WM_ is the mean value of the WM over the entire brain and *σ*_WM_ is the standard deviation of the noise. The factor of 1.2 raises the baseline of the WM signal closer to that of the GM signal. This is a very conservative threshold designed to avoid false positives. A typical patient case from the patient's group (with many lesions and a total WMH volume more than 10 mL) was chosen to analyze the receiver operator characteristic (ROC) curve to determine the high pass filter window size and *λ* which minimize the number of false positives. The segmentation results of the candidate lesions were labeled in 3D, and their distance from the ventricular CSF and their fractional anisotropy (FA) values were calculated. Here we use FA value to denote the shape character of the candidate lesion, using the moment of inertial structure tensor [15] instead of the diffusion tensor to calculate FA value. The candidate lesions signal intensity was weighted to calculate the eigenvectors in three orthogonal directions. Finally, candidate lesions were removed if they had either a very small distance to the cortical CSF (2.0 mm) in the sulci or a very thin prolate spheroid shape (high FA value over 0.6).

#### 2.2.5. Small Lesion Segmentation

The high frequency suppression step not only removes the GM boundary false positives, but also tends to remove small lesions (since they are intrinsically high spatial frequency in nature). Here, we define a lesion with less than 0.025 mL (100 pixels) as a small lesion. These can be detected using a higher threshold (1.2 · S_WM_ + 4 · *σ*_WM_) in the bias field corrected image. Then the distance from CSF, FA characteristic and ventricle information were utilized to remove the false positives.

#### 2.2.6. Lesion Volume Estimation

Since the edges of bigger lesions were removed in step 4, and only the highest components of the smaller lesions were kept in step 5, we applied a region growing algorithm (dynamic programming) [16] to return the candidate lesions to their actual size. From this point, the number of lesions and the size and intensity of information of each lesion can be measured.

#### 2.2.7. Categorizing the Lesions

Finally, the ventricle position information can be used to determine the category of the lesions. Those lesions with an edge that is less than 3.0 mm to the ventricles are classified as PVH while the rest are classified as DWMH.

### 2.3. Human Interfacing and Correction Schemes

The total number of lesions and the volume of the lesions were measured through manual segmentation to generate a gold standard for lesion number and volume. To assess the agreement between the gold standard and the proposed method, we used the similarity index (SI) measures:

(3) $$\begin{array}{c} \text{SI}=\frac{2\cdot \left(V_{\text{auto}}\cap V_{\text{GS}}\right)}{V_{\text{auto}}+V_{\text{GS}}}, \end{array}$$

where *V*_auto_ denotes the lesion area obtained from automated segmentation and *V*_GS_ is the gold standard obtained from the processor's manual segmentation (two processors reviewed each other). The SI value ranges from zero to unity, with zero for total disagreement and unity for a hundred percent agreement of the two methods. The algorithm was implemented in C++ and integrated into our in-house software SPIN (Signal Processing in NMR, Detroit, Michigan).

## 3. Results

**Figure 2:**
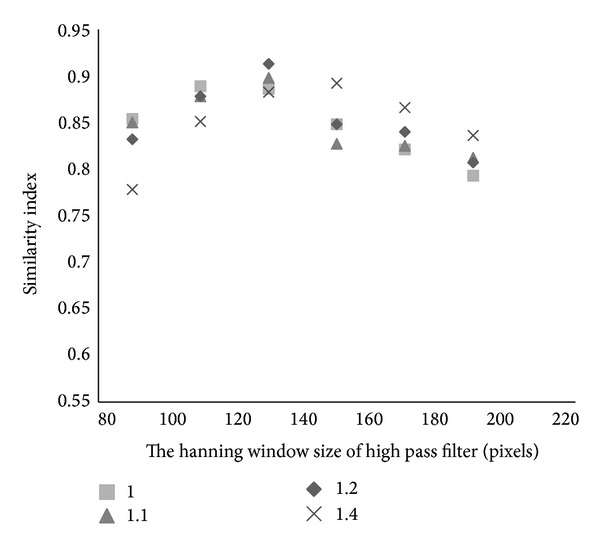
Receiver operating characteristic plot to determine the parameters of the high spatial frequency suppression filter.

**Figure 3:**
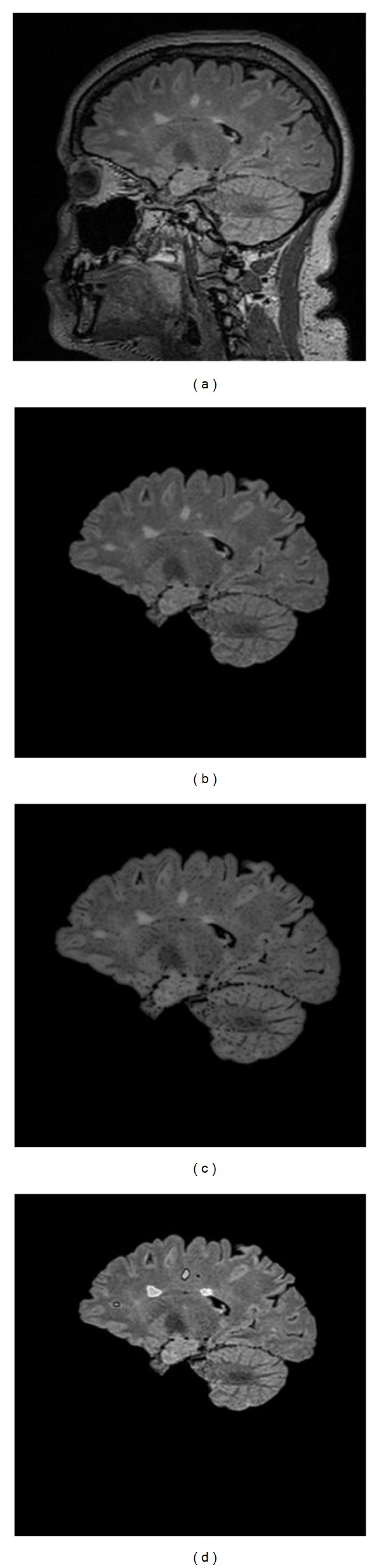
A 3D sagittal view FLAIR brain image. (a) Original images; (b) after skull stripping and bias field correction; (c) after high pass suppression of GM edges; and (d) the final segmentation of lesions and assignment of their locations (black contour for DWMH, white contour for PVH).

In order to evaluate the method as a whole, the high spatial frequency suppression parameters were first optimized. This was done using the ROC curve approach as shown in Figure 2 where we plot the similarity index as a function of both filter size and constant value *λ* used to enhance the reduction of high spatial frequency or GM edge information. These results suggest that the best choice of high pass window size is 130 pixels with a *λ* value of 1.2. With these variables fixed, the algorithm can be run automatically and compared to a manual segmentation approach. An example set of processed images is shown in Figure 3. The DWMH and PVH were denoted by black contour and white contour. The total processing time for each case takes less than 5 minutes (Intel i7 2.8 GHz, 8 G RAM).

**Figure 4:**
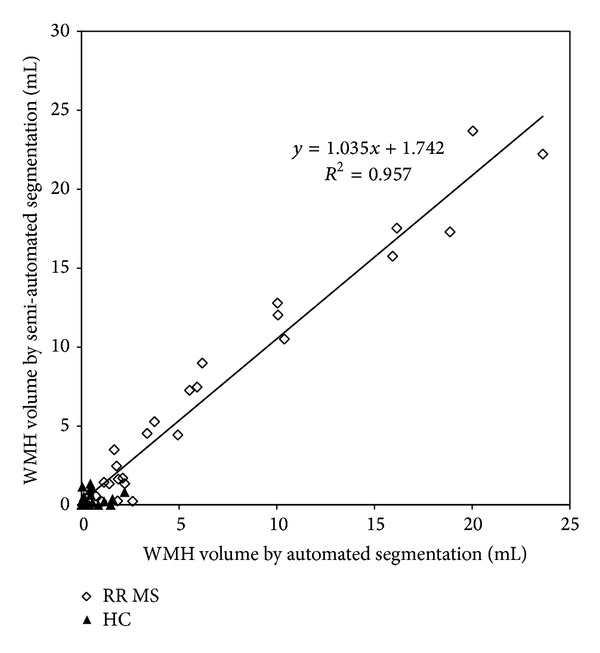
Correlation of the manual segmentation and automated segmentation methods. The open diamonds represent the MS patients and the solid triangles represent normal controls.

**Figure 5:**
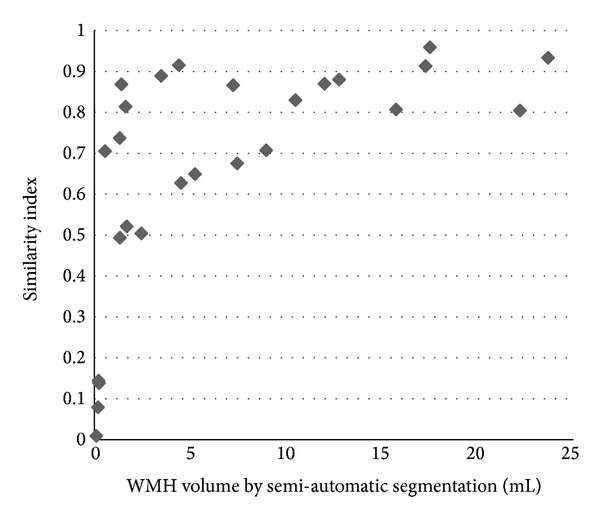
Similarity index for all 26 patients plotted over lesion volumes as derived from manual tracing.

The scatter plot of the WMH volume obtained using both the manual segmentation and the automated method is shown in Figure 4. The correlation between these two approaches in comparing WMH volumes for all 26 MS cases was found to be *Y* = 1.04 · *X* + 1.74  (*R* ^2^ = 0.96). Clearly, the age matched healthy controls show much less WMH than the MS patients. The results for the similarity index for the WMH volume reveal that the larger lesions were well detected by the proposed method (Figure 5). A review of three random cases showed that lesions as large as 0.15 mL could be missed if the contrast-to-noise in that area was poor but most lesions missed were closer to 0.012 mL.

## 4. Discussion

We have presented an effective method for automatic segmentation of WMH using 3D T2 FLAIR images. FLAIR imaging is one of the most popular protocols for MS. In particular, it has been shown that 3D FLAIR reveals more lesions than conventional axial T2WI [17]. Using 3D FLAIR also avoids the need for registration or collection of different datasets such as T1WI, T2WI, and spin density WI when brain volume and lesion load are needed. This algorithm is easy to set up for batch processing of data collected from the same site with the same imaging parameters for each patient. The output of this algorithm includes lesion number, volume, and lesion type such as PVH or DWMH. A particular strength of this approach is that all the image processing steps are integrated within a single interface, with a clear work flow interface that allows the user to change options as desired. Several semiautomated and manual assist methods are also provided to allow the user to override some of the automated components.

Other methods to segment WMH have been presented in the literature [5]. Most other approaches require more than one type of imaging technique. For example, Khayati et al. [18] used 2D FLAIR images with low resolution and the partial volume effect reduced the accuracy of the segmentation results. More recently, Simões et al. [6] developed an automatic segmentation using only 3D FLAIR images but required BET and FAST for the preprocessing and 3D Slicer for evaluation. To the best of our knowledge, our approach is the only integrated software tool using only 3D FLAIR images.

The choice of *λ* also had an effect on the scaling factor used to raise the WM baseline level to that of the GM when the threshold *p* · S_WM_ + 4 · *σ*_WM_ is used. For *λ* = 1.2,* p* was 1.2 while for *λ* = 1.4,* p* was 1.0. We chose the former based on its higher similarity index. The factor of 4 · *σ*_WM_ appears in the thresholding for small lesions because the background variation of WM was such that the usual choice of 2 · *σ*_WM_  or  3 · *σ*_WM_ still captured false positives.

This study has several limitations. First, the false positives caused by the gray matter boundaries are problematic. We have made major strides in dealing with this by using our high pass filter approach, but there still remained a few false positives that escaped automatic removal. Figure 5 shows the relationship between the similarity index and the candidate WMH volumes. The cases with total lesion volume over 0.5 mL achieved the highest similarity index values (average SI = 0.77). Second, the optimal data processing parameters are dependent on the imaging parameters. If the FLAIR imaging parameters are changed significantly, it may be necessary to redo the ROC analysis to find the optimal parameter settings. Third, we use the location of the lesions relative to CSF in the sulci to remove false positive lesions from GM. However, some of these cannot be easily detected. In addition, the lesions in the GM may also be falsely removed, if they are too close to the CSF. The type of lesion was classified by its distance from the nearest ventricle edge. When the ventricles could not successfully extracted then the lesions could not be classified correctly.

In conclusion, we have developed an algorithm that automatically estimates the volume and category of WMH using a 3D T2 FLAIR series of images. The automated quantitative algorithm has been shown to correlate well with the manual segmentation result. This approach should make it easier to study how the lesion load relates to other factors in MS and to monitor the number and volume of lesions over time.
